# A Qualitative Study on Emotions Experienced at the Coast and Their Influence on Well-Being

**DOI:** 10.3389/fpsyg.2022.902122

**Published:** 2022-06-10

**Authors:** Marine I. Severin, Filip Raes, Evie Notebaert, Luka Lambrecht, Gert Everaert, Ann Buysse

**Affiliations:** ^1^Flanders Marine Institute (VLIZ), Ostend, Belgium; ^2^Centre for the Psychology of Learning and Experimental Psychopathology, KU Leuven, Leuven, Belgium; ^3^Department of Experimental Clinical and Health Psychology, Ghent University, Ghent, Belgium

**Keywords:** restorative environments, blue space, emotions, well-being, interpretative phenomenological analysis

## Abstract

Coastal environments are increasingly shown to have a positive effect on our health and well-being. Various mechanisms have been suggested to explain this effect. However, so far little focus has been devoted to emotions that might be relevant in this context, especially for people who are directly or indirectly exposed to the coast on a daily basis. Our preregistered qualitative study explored how coastal residents experience the emotions they feel at the coast and how they interpret the effect these emotions have on them. We conducted semi-structured interviews with a purposive sample of eight Belgian coastal residents aged 21–25 years old. The interviews were analyzed with the approach of interpretative phenomenological analysis. Five superordinate themes were identified and indicate that, for our participants, the coast represents a safe haven (1) in which they can experience emotional restoration (2), awe (3), and nostalgia (4). These emotional states are accompanied with adaptive emotion regulating strategies (5), such as reflection and positive reappraisal, that may facilitate coping with difficult thoughts and feelings. Our study demonstrates the importance of investigating specific emotions and related processes triggered at the coast and how these could contribute to the therapeutic value of the coast.

## Introduction

Throughout recent years, there has been an increasing amount of research demonstrating multiple health and well-being benefits from exposure to blue spaces, i.e., natural environments that feature water (Gascon et al., 2017; White et al., 2020), with a particular focus on coastal environments. Although the coast has been visited by people for leisure, relaxation and thalassotherapy already since the 18th century (Verhaeghe, 1843; Strange, 1991), it is only until recently that there is empirical evidence of the relationship between the coast and well-being. Much of this evidence has demonstrated that residential proximity to the coast is associated with a better physical and mental health in comparison to residents living inland (Garrett et al., 2019; Hooyberg et al., 2020).

Various factors have been suggested to contribute to the well-being benefits of living near the coast. For example, coastal proximity is positively associated with the likelihood of achieving recommended guidelines of physical activity a week (White et al., 2014). Coastal environments are also seen as areas that boost social interactions (Ashbullby et al., 2013) and spending time with family and friends is stated as a key motivation for visiting the coast (Elliott et al., 2018). Furthermore, several environmental factors can explain the positive effects of the coast on well-being such as temperature regulation (Völker et al., 2013) and sea spray aerosols (Van Acker, 2021). Finally, coastal environments are considered as restorative in terms of stress reduction (White et al., 2013) and restoration of depleted cognitive abilities (Gidlow et al., 2016).

Considering the suggested mechanisms underlying the relationship between the coast and well-being, research has hitherto mainly focused on physical, social, and cognitive factors whereas emotional factors remain ambiguous. To create a more comprehensive framework of how and why the coast benefits well-being, it is imperative to also consider potential emotional processes. Although various emotional processes related to the coast have been suggested by earlier studies (Bell et al., 2015), the exact nature of these emotions and their effect on our well-being remain unclear. One way to investigate these emotions is to look at how people experience their emotions when they are at the coast. Nonetheless, the majority of qualitative research on emotions at the coast were conducted with visitors or tourists (Willis, 2015; Jarratt and Sharpley, 2017; Pearce et al., 2017), and not coastal residents. Due to the direct or indirect exposure to the coast on a daily basis, we can question whether coastal residents experience similar emotional processes at the coast, or perhaps to a lesser degree. To fill this knowledge gap, the present study is therefore based on the following research question: how do coastal residents make sense of the emotions they experience at the coast and of how these emotions affect them?

### The Coast as a Therapeutic Landscape

The relationship between the coast and well-being can be set within the concept of *therapeutic landscapes*, defined by a landscape where “physical and built environments, social conditions, and human perceptions combine to produce an atmosphere which is conducive to healing” (Gesler, 1996, p. 96). In other words, a therapeutic landscape can be any place with physical and social features that enable physical, mental, and spiritual well-being, as perceived by the people engaging in it.

Blue spaces, and thereby coastal environments, have been investigated as therapeutic landscapes (Völker and Kistemann, 2013; Bell et al., 2015; Finlay et al., 2015; Foley and Kistemann, 2015; Satariano, 2019). These studies explored three key dimensions of therapeutic landscapes experienced at the coast, namely the physical dimension, the social dimension, and the symbolic dimension (Gesler, 2003). The physical dimension relates to the diverse set of activities that the coast offers and its associated benefits. Participants from the study of Bell et al. (2015) described that these activities can fulfill a desire for challenge and achievement and provide emotional and cognitive release. Swimming, for example, has been shown to have a beneficial effect in the treatment of chronic disorders such as rheumatism or arthritis (Foley, 2010) and is known to support the maintenance and recovery of health (Foley, 2017). The social dimension refers to the opportunities for planned or spontaneous social interactions at the coast, as well as a collective experience of nature that enables a sense of community and social identity (Finlay et al., 2015). In addition, the coast is considered to be of intergenerational value as it has been shown to be beneficial for all age groups, containing an atmosphere of play and relaxation (Ashbullby et al., 2013; Satariano, 2019). The symbolic dimension of therapeutic landscapes relates to the historical, cultural, and individual perceptions of the coast and its effects on well-being. For example, in Bell et al. (2015), perceptions of water as cleansing or purifying conditioned the participants’ experience with coastal waves and associated moods and emotions. Besides individual perceptions of the coast, different cultural notions and traditions most likely also influence how specific communities interact and experience the coast and its effects (Wheaton et al., 2020).

### Emotions at the Coast

One dimension pertaining to the therapeutic landscapes concept that has not been specifically looked into is the emotional dimension. Only a handful of studies explicitly focused on emotional processes linked to the coast (Willis, 2015; Jarratt and Gammon, 2016; Jarratt and Sharpley, 2017; Pearce et al., 2017). In these studies, two emotions are prominent, namely the emotions of awe and nostalgia. Awe is an emotion that occurs whenever one perceives something as larger than the self and when one feels the need for accommodation to assimilate it (Keltner and Haidt, 2003). It is an emotion that is characterized by feelings of a small self, in which one’s attention is less focused on the self (Shiota et al., 2007) and one’s personal concerns and goals become less significant (Piff et al., 2015). Typical stimuli that elicit awe include art, music, and natural landscapes with panoramic views (Shiota et al., 2007). The coast in particular appears to trigger awe, accompanied with a change in perspective and feelings of relaxation (Willis, 2015; Jarratt and Sharpley, 2017). In a study by Pearce et al. (2017), when asked about what inspires awe at the coast, participants replied with five different facets: marine fauna, aesthetics, ecological phenomena, vast geological landscapes and reflective moments.

The second prominent emotion, nostalgia, is characterized by a sentimental yearning for the past, and although historically considered to be an emotion of negative affect (McCann, 1941), it is increasingly being regarded as ambivalent, thereby containing both positive and negative affect (Sedikides and Wildschut, 2016). Nostalgic experiences are associated with redemption (Wildschut et al., 2006), whereby the narrative progresses from a negative standpoint into a positive one, e.g., taking the good out of the bad (McAdams et al., 2001). In relation to the coast, the study by Jarratt and Gammon (2016) showed that seaside visitors feel nostalgic during their visits to the coast, due to the unchanging and timeless nature of the coast, in contrast to the fast-paced modern world. In addition, nostalgia can be triggered by sensory input (Wildschut et al., 2006), in line with findings that tastes and scents can elicit autobiographical memories (Chu and Downes, 2000). The coast most likely provides sensory stimuli that can lead to nostalgia, such as the scent of the ocean (Reid et al., 2015).

Both awe and nostalgia are considered as complex emotions, namely that they are a combination of two or more emotions. Nostalgia, for example, can be regarded as an aggregate of sadness and happiness (Power, 2006) and awe can be perceived as a mix of fear and happiness (Arcangeli et al., 2020). Moreover, awe and nostalgia are shown to positively affect well-being. Within the context of eudaimonic well-being, which defines well-being with such concepts as autonomy, environmental mastery, personal growth, positive relationships, purpose in life, and self-acceptance (Ryff and Keyes, 1995), both emotions contribute to meaning in life (Routledge et al., 2012; Danvers et al., 2016). Nostalgia has also been shown to enhance self-esteem and social connectedness (Hepper et al., 2012; Reid et al., 2015). Within the context of hedonic well-being, considered as a state of positive affect and absence of negative affect (Kahneman et al., 1999), studies find that nature-induced awe is associated with life satisfaction (Anderson et al., 2018) and is negatively correlated with boredom (Severin et al., 2021). Nostalgia is also shown to buffer against boredom (Van Tilburg et al., 2013) and to generate positive affect (Wildschut et al., 2006). Considering the positive effects of awe and nostalgia on well-being, it is possible that experiencing these emotions at the coast contributes to the role of the coast as a therapeutic landscape.

An additional mechanism that can pertain to the emotional dimension but does not relate to an emotion *per se* is psychological restoration, defined as a renewal or recovery of depleted physical, psychological, and social resources (Hartig, 2004). More specifically, emotional restoration relates to feeling calm and relaxed as well as feeling refreshed and revitalized. Studies show that visits to natural environments, and particularly the coast, lead to greater emotional restoration (Hartig et al., 1991; White et al., 2013). A prominent theory related to this phenomenon is the stress recovery theory (SRT; Ulrich et al., 1991). The SRT posits that, compared to an urban environment, an environment with natural features facilitates positive affect—notably feelings of interest, pleasantness, and calm, and also reduces both physiological and psychological stress, due to an innate affinity toward nature (Ulrich, 1983). Aquatic features in particular seem to provide greater restoration in terms of relaxation and positive affect (Ulrich, 1981; White et al., 2010). Emotional restoration can therefore also be an important mechanism in in the relationship between the coast and well-being.

### Present Study

The present study aimed to provide an in-depth analysis of how coastal residents experience the emotions they feel at the coast and how they interpret the effect these emotions have on them. To do so, we conducted semi-structured interviews with a group of young Flemish adults who grew up near the Belgian coast and/or were currently residing near the coastline. This particular age group was chosen due to the multiple stressors young adults are exposed to (e.g., instability in work and relationships, transition to living apart from parents, identity exploration) and their resulting mental health implications (Arnett et al., 2014). We subsequently analyzed the interviews with the approach of interpretative phenomenological analysis (IPA; Smith et al., 2009).

## Materials and Methods

Our study’s aim, design, and analysis plan were preregistered on the Open Science Framework (OSF) registry before data collection.^1^

### Participants

In line with the principles of IPA, which will be discussed further, we recruited a purposive homogeneous sample of eight participants. The authors of IPA consider an acceptable sample size to be between four and ten participants (Smith et al., 2009). Inclusion criteria involved being between 18 and 25 years old and living near the Belgian coast. A description of the eight participants can be found in Table 1.^2^ All participants were from the region of Flanders and therefore the interviews were conducted in Dutch.

**Table 1:** Participant characteristics.

Participant pseudonym	Sex	Age	Living situation	Studies/work	Extra characteristics
Arne	Male	*MV*	Grew up and is living near the coast	Student	Diagnosed with autism spectrum disorder
Emma	Female	22	Grew up near the coast and lives inland in student room	Student	
Justine	Female	22	Grew up near the coast and lives inland in student room	Student; on Erasmus in another country	
Noa	Female	22	Grew up and is living near the coast	Temporarily employed; seeking work	Diagnosed as a highly sensitive person
Anna	Female	22	Grew up near the coast and lives inland in student room	Student	
Louis	Male	22	Grew up near the coast and lives inland in student room	Student	
Laura	Female	25	Grew up inland and lives near the coast	Employed	
Victor	Male	21	Grew up and is living near the coast	Student	

MV stands for “missing value.” The participant Arne did not inform us his age.

### Procedure

Two participants were recruited *via* advertisement of the study in a university school in Ostend, Belgium, whereas the rest of the participants were recruited *via* word-of-mouth. Participants were notified of the study’s inclusion criteria, its aim to explore how coastal residents experience living near the coast, and the registration procedure. Upon registration, participants were asked to fill out an informed consent form. Interviews were then conducted *via* Skype, during the months of October to December 2020, to respect measures against the COVID-19 pandemic at that time. The interviews lasted between 40 and 60 min and were digitally recorded, transcribed and pseudonymized. After the interview, participants received a financial compensation of 15 euros (approximately 17 USD). The study was approved by the ethical committee of the Faculty of Psychology and Educational Sciences of Ghent University.

The semi-structured interviews followed a predetermined interview guideline that consisted of 13 open questions (available at https://osf.io/v2cax/). Participants were first asked to introduce themselves and provide insights into their daily routines to put them at ease. They were then asked to describe their relationship with the coast and the impact of living near the coast on their daily lives. Finally, they were asked about the specific emotions they feel at the coast and what effect these emotions have on them. Extra prompts were prepared and sometimes used to facilitate further discussion, such as asking whether the emotions are accompanied with particular physical sensations or thoughts. This guideline helped to steer the conversation toward the questions of interest. Nonetheless, we aimed to leave space for participants to share their story and to bring up new areas that were not considered before. This type of interviewing enables more flexibility to explore participants’ interests or concerns and facilitates a rapport between the researcher and participants (Smith and Osborn, 2015). In order to familiarize themselves with this kind of interviewing, each researcher conducted a pilot interview with an external person that was unaware of the study’s aim.

### Qualitative Data Analysis

The transcribed interviews were analyzed with the use of IPA. The aim of IPA is to investigate in a detailed manner how participants make sense of a specific phenomenon or experience and the meanings that they attach to it. IPA is thus an ideal approach for our study as it focuses on the lived experience of a particular group and is best suited for research questions that are understudied (Peat et al., 2019). IPA is constructed around three principles: phenomenology, hermeneutics, and idiography. Based on phenomenology, IPA researchers strive to look into participants’ subjective experience rather than make an objective statement of the experience itself. However, the principle of hermeneutics shows that access to this experience depends on participants’ interpretation of it as well as the researcher’s interpretation of the participants’ interpretation. In opposition to the nomothetic approach, the last principle, idiography, dictates that IPA researchers prioritize an analysis on a case-by-case basis and therefore avoid making premature general claims for larger populations. Purposive sampling is thus used to find a “more closely defined group for whom the research question will be significant” (Smith and Osborn, 2015, p. 56).

In our study, we employed IPA using a step-by-step procedure demonstrated in Table 2 (Smith et al., 2009). Each interview was analyzed individually, before being compared with each other. This individual analysis consisted of first familiarizing oneself with the interview by making descriptive, linguistic, and conceptual notes. An example of a linguistic note was to notice a participant anthropomorphizing the sea (“that is something that the sea never asks”), which indicated perceiving the sea as a powerful entity. We then identified emerging themes based on connections among these interpretative notes, such as “the coast representing home,” or “fascination towards the sea.” These subthemes were subsequently clustered into five or six overarching themes that conceptually differ and that are prevalent in the interview. Each theme or subtheme is illustrated by one or several direct quote(s) of a participant. This process is repeated for each participant, with the conscious effort to put aside the analysis of the previous cases, known as “bracketing” (Peat et al., 2019). The final step involved a group analysis to construct a final table of superordinate themes, based on the themes of each participant. Some themes were found for every participant and thus shifted into superordinate themes, while other themes were clustered to create one new theme. This final table of themes represents the findings of our study.

**Table 2:** Step-by-step procedure for interpretative phenomenological analysis.

1. Read and reread transcript of interview and make descriptive, linguistic, and conceptual notes.
2. Develop emergent themes based on the notes and the transcript.
3. Cluster themes together to create a table of overarching themes.
4. Repeat steps 1–3 for each participant, with the conscious attempt to minimize the influence of the analysis of the previous interviews.
5. Compare the individual table of themes with each other: similarities, differences, and patterns between transcripts are noted.
6. Construct a final table of superordinate themes that stems from all participants and includes individual exceptions.

While conducting IPA, we aimed to respect reflexivity, which is the process of being aware of how the perspectives and beliefs of the researchers might influence the analysis procedure (Dodgson, 2019). The transcripts of the interviews were analyzed by two Master students (EN and LL) and one doctoral student (MS), under the supervision of a professor (AB). Although the authors enjoy visiting the coast, neither of them has or has had a permanent residency near the coast. One of the authors (EN) did live in a coastal city while practicing surfing for 6 months and experienced this very positively. Nonetheless, the analysis process was done in collaboration between the authors. Several meetings took place in which the authors discussed the findings. The first transcript was analyzed by all the authors, with the joint construction of its table of themes. The remaining transcripts were analyzed separately, and the final table of themes was subsequently set up together. In this way, the interpretation of the interviews could not be based on an individual researcher perspective and conscious effort was made to not impose personal feelings toward the coast during these collaborative discussions. According to the framework of Yardley (2000), rigor was achieved by implementing this reflective process, the use of bracketing and pilot interviews, and ensuring a sample that was appropriate for our research question.

## Results

The IPA results of our semi-structured interviews consist of five superordinate themes: emotional restoration, awe, nostalgia, emotion regulating strategies, and the coast as a safe haven. The themes are presented in the following sections. We would like to emphasize that the interviews and analysis were conducted in Dutch and therefore all quotes from participants have been translated to English.

### Emotional Restoration

The coast appears to have an emotionally restorative effect on our participants as they all described feeling calm or revitalized when visiting the coast. This effect was however expressed differently by each participant. Justine, for example, spoke of “feeling reborn” when she is at the coast and claims that spending time at the sea makes her feels more “recharged” and is “more effective than watching a TV series for 20 min.” For Noa, being at the coast calms her down and brings her “peace of mind.” Some participants compared the coast with urban environments, such as Louis who stated that he feels calmer at the coast “because in any case in a city, there is often more movement, more people, and more stimuli.” The feeling of calmness appears to come from the availability of open space and the accompanied opportunities to do physical activity: “by the sea you can just walk or cycle meters on the beach, and you just immediately calm down a bit” (Louis). For other participants, simply seeing the coast brings them a feeling of calmness.

Emma: “I often feel that I only really relax when I have seen the sea. Then, I feel it so almost physically that something falls off of me or something …, even if it is sometimes only for five minutes just having seen the sea that does a lot, yes that really means a lot.”

The restorative effect of the coast can be especially prominent during certain stressful moments. For example, Anna typically enjoys relaxing near the sea during exam periods. For Emma, going to the coast “always helps” when she “feels a lot.” She adds that although this does not solve anything, “it does help because it calms me down.” Furthermore, in comparison to the other participants, Laura indicated that feeling calm at the coast happens only occasionally for her, notably when she is alone or “when it has been a busy day at work or you have a headache or something.” The feeling of calmness that the coast brings also seems to decrease during the summer due to increased tourism. Laura, for example, expressed that “in the summer I have in any case the opposite I think, then the sea makes me much more nervous than [name of city] would make me.” Tourism therefore seems to reverse the soothing effect of the coast for several participants.

### Awe

During the interviews, almost all participants spoke to some extent about an overwhelming effect of the coast on themselves that seems to reflect the emotion of awe. A contrast emerged between awe with a positive valence and awe with a negative valence. Positive awe was expressed by some participants as a fascination for the sea and its natural elements and a respect for its power and grandeur: “the sea is also very fascinating because when you stand by the sea and you listen to the noise that the sea makes, you know that there is an enormous power within it.” (Arne). The interplay between different natural elements and sensory stimuli could make a deep impression on some participants. Justine, for example, was particularly impressed by the vastness of the coast; she stated that the sea is “such a large body of water that we do not control.” The dynamic aspect of the coast was also referred to by some participants, such as the infinite repetitive motions of the waves, the “color changes” of the coast as well as the tidal changes, “the ebb and flow.” Several participants also mentioned the particular beauty of other natural phenomena of the coast such as the “blue algae in the waves,” “waterspouts,” “snow on the beach,” and “beach insects.” Furthermore, the power of the sea was an important element in triggering awe for some participants. A feeling of adrenaline and excitement was described when faced by a stormy sea.

Justine: “When it storms, I love to go for a walk on the beach. Even though they often advise against that (laughs), but uh, I think it’s really cool to see that – yes, I think that’s something really overwhelming, the sea.”

Louis, who works as a beach lifeguard, also described an awareness and appreciation of the dangerous elements of the sea. For Arne, it is the “science” behind the sea that fascinates him, such as the sea’s regulation of temperature, as well as the battle between the sea and manmade constructions at the coast, in which “construction sometimes wins, sometimes loses to nature.”

In contrast to feeling positive awe at the coast, certain participants expressed awe in more negative terms, particularly toward the mystery and the grandeur of the sea. Victor, for example, finds it “frightening” to “go really deep into the water” and Laura expressed displeasure of not being able to “see what’s happening at your feet” when she’s in the water. Both participants also conveyed fear toward the grandeur of the sea, especially during a storm.

Victor: “I find it scary sometimes, because it's so majestic, it's so big, and then when there's a lot of wind and high waves and stuff, I can be impressed by that, and that can instill a certain fear in me.”

This sort of negative awe can perhaps be due to not being able to fulfill the need for accommodation. Indeed, certain participants described trying to understand the grandeur and mystery of the coast. For example, Arne tries to comprehend how the strength of the sea does not break the pier: “how in God’s name is it possible that the pier and the railing all remain standing when those waves crash into them.” Victor also displayed his reflections over the history of the coast and what lies beneath the horizon. Nonetheless, Louis depicted an example of how negative awe can lose its negativity through frequent exposure to the coast:

Louis: “if you often go into the sea and you become a bit used to what those dangers are, or you know what to do and such, … you do get a little less frightened or something, and it becomes less threatening.”

Louis was therefore able to adapt his mental schemas concerning the threatening elements of the sea and thereby feel less fear.

Moreover, the emotion of awe at the coast appears to have certain effects on the participants. These effects could be in the form of physical sensations or specific feelings that portray an emotional impact upon the participant.

Victor: “you can get a shiver down your spine … if you’re so impressed by that situation or something. Or I’ll then get a lump in my throat, or there can be once a (laughs) – a tear rolling down …, I can be caught off-guard like that yes.”

In addition to these physical sensations, awe also seems to lead to a feeling of small self, as stated by Arne: “the grandeur, the greatness of the beach, of nature, of the dike … and then you just stand there as a small, small dot, yea then you just feel very humble and from there you start thinking about life.” Furthermore, for other participants, the emotion of awe facilitates a feeling of calm, such as for Laura who “calms down” when she “can enjoy the fact that it [the coast] is beautiful.”

### Nostalgia

In addition to awe, the coast seems to generate nostalgia in participants by evoking important memories that were created there. All participants described unique memories of the coast throughout their lives. The majority spoke positively of these memories with a nonverbal behavior that portrayed pleasure and gratitude (e.g., laughter, smile). Nonetheless, some participants recalled memories that appeared to be negative. Anna, for example, recounted the death of a family friend who used to take photos of the coast and she thereby sometimes thinks back to that event while at the coast. She further expressed how this makes her feel sad but that “it’s ok” and that being at the coast makes her feel more “calm” because the “sound of the sea is also calming.” Thinking back to this memory is therefore bittersweet as Anna experiences both sadness and a “beautiful” feeling.

A great deal of the memories described by participants were socially-oriented. Examples involve activities with friends and family such as parties in the evenings, walks on the beach, summer vacations, games and watersports. However, some of the memories were not focused on social features and were instead directed toward natural phenomena at the coast. For example, Arne and Noa longed for the winter, and more specifically, snow on the beach.

Arne: “the best memory was when it still snowed at the coast …, that the beach was sometimes a bit white and so was the dike, yea just these idyllic pictures …, yes these are the memories I cherish the most about the dike and the sea.”

Additionally, storms at the coast seem to trigger important moments that involve challenging one’s own boundaries. Noa, for example, talked about an enduring memory, when she swam in the sea with her younger brother on a stormy evening, and emphasized the “rush” and adrenaline that she felt. Other memories associated with the coast that were not socially-oriented were memories of daily routines at the beach such as walking the dog or watching the sunset. Walking at the coast seems to arouse nostalgia in Arne when he thinks about all the “beautiful moments” he spent with his dog at the beach and he wonders “how much longer they can still enjoy” these moments. Justine also expressed missing the coast and her routines there as she was on an Erasmus exchange in a city with a strict lockdown and had no access to the coast at the time of the interview.

The coast appears to play an important role throughout the lives of the participants as they described enduring memories of the coast from their childhood and teenage years. For example, Laura mentioned how she, as a child, did things like “every child”: “sit on the beach and sell those flowers and go look for shells.” Teenage years were then described as “pretty fun days, young teenage days …, days playing with friends at the beach, playing in the sea” (Noa). Many of these memories were linked to summertime at the coast and its “carefreeness,” which generated nostalgic feelings in several participants, as Emma mentioned missing “the whole summer atmosphere.” However, unlike the rest of the participants, Arne had the least memories of the coast in his childhood. He has autism spectrum disorder (ASD) and is quickly overwhelmed when there are a lot of people; because of increased tourism, he therefore could not spend much time on the beach when he was a child and instead went more often to parks.

Apart from creating important memories, the coast also seems to trigger the reminiscence of these memories. Victor, for example, explained how being at the coast makes him remember a special moment with his friends during high school:

Victor: “I remember that moment sometimes when I go to the beach in the summer, it reminds you of friendship and of the old days. It is unconsciously so that many things directly or indirectly have something to do with the beach, with the proximity of the sea.”

Certain sensory stimuli of the coast were considered to evoke memories such as the scent of the beach that reminds Anna of the summer. For Arne, this effect of reminiscence seems important as he indicated that he “would still visit certain places regularly just to reminisce” if he no longer would live near the coast. Justine also mentioned that the many beautiful memories of the beach “play a part in the fact that she likes being there.” Various feelings seem to accompany the reminiscence of memories at the coast, such as a “feeling of happiness,” “a little homesick,” a feeling of “loss,” or a feeling that time goes quicker.

### Emotion Regulating Strategies

In the interviews, various emotion regulating strategies were depicted, either in relation to the emotions mentioned above, or independently associated with the coast. Although there is clear overlap between them, we describe them separately below.

#### Reflection and Meaning-Making

Several participants expressed that the coast is a place where they could stand still and reflect on life, problems, and emotions: “the big questions of life then start to play out” (Arne). For Justine, it is “one of the few moments” that she thinks about her emotions. Victor also benefits from being able “to reflect on his feelings and his emotions” at the coast, because he “maybe sometimes does that too little in his daily life.” He specifies that he is “not consciously” thinking about specific things but that “things are always going through his mind.” In contrast, for Emma and Justine, the coast can also just be a place for them to think of nothing.

Arne emphasized that the possibility of contemplation during his “long walks” on the beach is important to him as it helps him to sort things out and to go over the “mistakes” he might have made during the day, often in the context of his ASD. Regarding these mistakes, Arne displayed signs of meaning-focused coping through acceptance as he says: “I made them [the mistakes] but I’ll make sure I do not make them twice.” The self-reflection that the coast offers can therefore potentially help Arne to open up to unprocessed feelings, such as guilt, shame, or frustration. Another sign of a meaning-making process is the humility and vulnerability that Arne feels when he experiences awe. As he realizes that “we are actually nothing compared to the sea,” he “tries to build a philosophy in his life” in which he is humble both to nature and to other people. Other participants also described meaning-making in terms of putting things in perspective, which helps them to focus on what is truly important to them and also helps to not keep stressing for the less important things in life.

Emma: “I also just realize more what I have, because I can be stressed about things, but in the end it’s not all that important as long as you … are healthy and as long as your family is okay then, I don't know, then I can fail as many exams that I want, but that just doesn't really matter.”

#### Letting Go and Exteriorizing Emotions

Some participants indicated how they can let go of their emotions at the coast. Two participants alluded to coastal elements facilitating this process. Noa, for example, mentioned “being able to let go of everything for a while” and her emotions could “literally slide into the earth or blow with the wind.” Emma also talked about “how it all blows away from you” with reference to the wind at the coast. In relation to this, Laura indicated that the coast was the best place for the Dutch practice called “uitwaaien,” which means to go outside in windy weather with the aim to clear one’s mind.

In addition to letting go of emotions, the coast also helps Justine in letting go of overthinking and makes her become more aware of the present moment: “everything else that goes on in your life, then I forget that and then I’m just … one with nature.” In particular, Justine expressed the importance of the coast at a time when she had to make a crucial decision, stating a constant need to be “preoccupied” with this decision, but being at the beach gave her the permission to temporarily let go of the weight of this decision. The coast therefore offers some participants the chance to get a breath of fresh air and to thereby let go of certain (often difficult) thoughts, feelings or emotions.

The coast was also considered to be a place where emotions could be exteriorized for certain participants, functioning thereby as an “outlet.” Noa spoke most explicitly about this when she stated that the coast is a place where she could express her emotions, including negative emotions: “sometimes I can really let go of my anger or my sadness, so to speak, so that it can really get out.” To exteriorize these emotions, Noa performs actions such as “screaming, crying, or singing.” This exteriorization stops her head from “spinning” and has a positive effect on her: “it calms me down, gives me peace of mind, gives me sometimes just the energy, to keep going.” The use of the coast as an outlet helps Noa to move forward, so that she “no longer has to think about it” and instead think: “tomorrow is a new day.”

#### Seeking Experiences of Peak Flow

The use of the coast as means of stimulation and seeking peak flow was demonstrated in two participants. Noa, for example, described at length how she uses the coast to pursue a “rush.” During her childhood, the coast gave her a place to “romp,” or in other words to play around in a lively manner, and to “seek out adrenaline rushes.” As Noa grew older, she started to do watersports such as kitesurfing, with which she would seek out “the challenges” and the “combination of wind and sea to then get the feeling thereof.”

Noa: “Feeling for me is really just being able to feel with all the sensors that I have without being directly overwhelmed, so in nature for me there is always a sort of vibe, a fixed wavelength … with which I can surf along. That also gives a secure peace of mind or a mental calmness.”

This type of experience can be referred to as an experience of peak flow. Noa related this experience to her being a highly sensitive person and thereby demonstrated that doing kitesurfing, or “moving flexibly her legs on the sea, absorbing these shocks,” helps her cope with her high sensitivity.

Arne also displayed how the coast is a place that positively stimulates him. As he described the fascination he feels when he sees the water hit the pier during a big storm, this does not negatively overwhelm him such as other stimuli in his daily life. On the contrary, he actively seeks out this stimulation as he explained how one time during a storm, he climbed over the fence that was used to close off the pier, to look at the water. At that time, he was “really excited” and found that “very pleasant.” Both Noa and Arne thus experience difficulties with processing stimuli but named the coast as a place where they can feel safely stimulated.

### Coast as a Safe Haven

The coast was described by participants in various ways as a safe haven, or in other words, a place where they could feel at ease and feel safe to experience emotions and vulnerability. This was conveyed by three different facets that are described below.

#### Respite From Daily Life

Several participants expressed how the coast provides them respite from the hustle and bustle of their daily lives.

Justine: “I find that with many other things in our lives, it is always very busy …, and by the sea, or the dunes, I find a place where you can once in a while be somewhat stimulus-free and that you return to a moment of zen.”

Coupled with this, Justine described how there is an absence of pressure and expectations at the coast. In her daily life, she feels like she “has to be in a hurry,” that she has things to do and things she should not forget, whereas the coast offers her “some more time to think” and enables her to relax. Arne also mentioned that there is an absence of social pressure and norms at the coast: “by the sea you have time, there is no one who says ‘you have to walk faster’, there is no one who says ‘you have to do this, you still have this much to do’.” Even the feeling of time itself can be absent at the coast, as Noa refers the coast as a “timeless place” and does not feel the need to look at the time when she is there.

Beyond providing respite, the coast also generates a sort of disconnection from reality for various participants. For example, Victor recounted how during his daily drive along the coastal road he can become so “lost in his thoughts” that he does not notice how far he went until he stops at a traffic light. This type of disconnection from reality was described by Emma in relation to the dunes, as she stated that the dunes make one feel really cut off from the rest of the world, in comparison to coastal cities that only have a dike.

#### Feeling of Safety and Freedom

An important aspect of the coast as a safe haven is the feeling of safety and freedom that the participants reported experiencing there. In terms of safety, Emma described the sea as a “very safe environment where she feels very good.” Other participants also mentioned “feeling at ease” at the coast, with Justine adding that she “never feels alone,” she “just always feels very reassured by the sea.” This sense of safety could come from the familiarity that participants have in relation to the coast. Some participants expressed how the coast is, and always will be, there for them, as Arne said: “when you are happy, when you are sad, you can always go to the beach.” Justine stated that even when she does not go to the coast, “just the fact that she knows it’s there” is reassuring for her. Additionally, the coast represents “home” for some participants, like for Arne who described the sea as his “homeland.” Justine even went so far to say that she “identifies with” the sea. It is therefore more likely that this familiarity with the coast explains the feeling of safety that participants feel there, rather than the coast itself. Anna even linked the two together by saying: “a sense of safety, yes, just a familiar environment actually.”

In addition to creating a feeling of safety, the coast also represents a place that facilitates emotions and vulnerability. For example, Noa indicated that she and her parents use the coast to have difficult conversations with each other. The coast is thus a setting that makes these conversations easier to have for Noa. Furthermore, Arne expressed how in his daily life, he feels he needs to be as rational as possible, but at the coast “he starts to become emotional,” by reflecting on life and “allowing the peace that the sea indirectly gives you.” This is the “absolute opposite of the feeling” he has during the day. An element that may support this effect on Arne is the simplicity and predictability of the sea, such as the infinite repetition of its waves. The outside world may feel unpredictable to Arne, because of his ASD, and experiencing the natural predictability of the coast may help release the pressure he feels during the day: “The sea that runs its course there, comes and goes, comes and goes. The simplicity.”

Some participants expressed a sense of freedom that they feel at the coast. For example, Louis described the coast as providing the freedom to “do what you want.” This sense of freedom appears to have been accentuated throughout the COVID-19 pandemic for most participants. This could be due to the easy accessibility of a wide-open space, as pointed out by Victor: “we were always lucky here that the sea was close by and the beach is vast, it is large, you can walk there with many people at the same time, without walking on each other’s feet.” Victor also mentioned that due to the diversity of the coast, with the beach, the dunes, and the forest, “you do not have to do the same tour everyday,” and this “played a positive role” on him. The coast was therefore perceived as an “extra freedom” during the first-wave lockdown, where the participants could walk or exercise.

#### The Opportunity to Be Alone

The opportunity to be alone at the coast also contributes to the representation of the coast as a safe haven as it is appreciated by several participants and appears to facilitate the emotion regulating strategies triggered there. For example, Noa and Arne explicitly seek out the coast when there are as few people as possible, as Noa “enjoys the emptiness when there are not many people on the beach” and Arne “just loves when you look over the length of the dike, that you really do not see anyone.” Being alone at the coast may help them cope with the overstimulation that they experience in their daily life. Other participants also mentioned the calming effect of having a “moment for themselves” (Justine), with Louis stating that it is especially “when he’s alone” that “he can really feel at ease.” Additionally, although Laura rarely visits the coast alone, she did indicate that the calming effect of the coast was mostly present when she walked alone.

## Discussion

Our qualitative study presents five superordinate themes that represent the emotional experiences generated at the coast for eight Belgian coastal residents.

### Emotional Restoration

The first theme relates to emotional restoration, as all participants reported feeling calm and/or revitalized when they were at the coast. This is in line with findings of a meta-analysis demonstrating a positive association between larger amounts of blue space within a geographical area and markers of restoration such as stress, anxiety, and depressed mood (Georgiou et al., 2021). For our participants, this feeling of calmness and peace of mind was linked to various coastal elements (e.g., wind, water, open space) and the opportunities for physical activity. This supports the claim that physical activity at the coast enables restoration, as emphasized by the Blue Gym initiative (Depledge and Bird, 2009). The emotional restoration experienced by participants also aligns with the stress recovery theory of Ulrich et al. (1991), as they stated feeling calmer at the coast than in urban environments. Nonetheless, coastal tourism appears to weaken or even reverse the effect of emotional restoration. This issue is referred to in the study by Bell et al. (2015); however, it remains understudied in the current literature. Further research is definitely needed to assess the impact of tourism on the restorative qualities of the coast.

#### Awe

The second theme that emerged throughout the interviews was the emotion of awe experienced at the coast. Participants described sensations and perceptions that are compatible with the definition of awe given by Keltner and Haidt (2003). The coast is perceived as larger than one’s self, which leads to a need for accommodation in some participants. The power and the grandeur of the sea appear to particularly trigger this perception, but other coastal elements such as the cyclic dynamic of the waves or the mystery beyond the horizon also seem to generate awe. In the study of Pearce et al. (2017), elements that were found to inspire awe at the Australian coast, such as its aesthetic beauty, tidal fluctuations, and vast landscape, were also reported by our participants for the Belgian coast.

Participants who described being unable to comprehend these awe-inspiring phenomena experienced a more negative type of awe with feelings of fear and anxiety. The valence of awe was indeed expressed dimensionally in our interviews and ranged from a fascination toward the power of the sea to unrest or fear toward the potential danger or mystery of the sea. Awe was therefore linked with both positive and negative affect, supporting the notion that it is an ambivalent emotion (Moss and Wilson, 2015). One of our participants demonstrated that frequently interacting with the sea allowed him to be less scared of it. Negatively valenced awe could thus transform into a positive fascination if one were to be more knowledgeable and familiar with the sea, allowing to successfully fulfill one’s need for accommodation.

An alternative concept that can be linked to these feelings of fear is the experience of the sublime. According to the philosopher Burke (1757/1990), this emotion is triggered by aesthetic stimuli that convey both power and obscurity, thereby inducing elements of threat and fear. Awe and the sublime are viewed as closely related, with studies suggesting that the sublime can be conceptualized as a threat-based variant of awe (Gordon et al., 2017; Arcangeli et al., 2020). However, recent studies call into question the importance of the fear component in the sublime, with one study demonstrating that awe and the sublime are associated through the dimensions of small self and connectedness instead (Clewis et al., 2021). Further research is needed to clarify the relationship between the sublime and awe and the extent to which the sublime can be linked with experiencing fear at the coast.

Other characteristics of awe appeared in our interviews and can be linked to the facets of awe in the Awe Experience Scale (AWE-S; Yaden et al., 2019). One of these facets is self-diminishment, which refers to the feeling of a small self, and was conveyed by one of our participants, Arne. Self-diminishment has been found to mediate the effect of awe on prosocial behavior (Piff et al., 2015) and humility (Stellar et al., 2018). Our study is consistent with these findings as Arne described feelings of small self that leads him to feel humble toward both nature and other people. Another facet of awe from the AWE-S that was described by our participants was the physical sensations accompanying the experience of awe, such as the “shiver down your spine” described by Victor. Moreover, one aspect of awe that is not included in the AWE-S, but that was expressed by Arne, is the fascination toward the science behind the sea. Studies have indeed shown that awe is positively associated with scientific thinking, to help fulfill the need for accommodation (Valdesolo et al., 2017; Gottlieb et al., 2018). Overall, the experience of awe at the coast reported by our participants is in line with previous studies demonstrating nature, and more specifically the coast, as an elicitor of awe (Shiota et al., 2007; Anderson et al., 2018; Ballew and Omoto, 2018).

#### Nostalgia

The third theme identified in the interviews refers to the experience of nostalgia at the coast. This experience is characterized by the coast evoking important memories that were created there throughout participants’ lives. The qualitative study of Jarratt and Gammon (2016) presents similar findings as their participants described a nostalgic connection with the coast, primarily through the recollection of childhood memories. It is interesting to note the similarity between the present study and the one of Jarratt and Gammon (2016), considering the age difference (their participants were aged between 55 and 74 years old). This similarity is however in line with previous findings showing that proneness to nostalgia follows a curvilinear trend, namely that it peaks in younger (under 30) and older (over 75) adulthood, (Hepper et al., 2021). This is possibly due to the importance of nostalgia in times of transition (Sedikides et al., 2015), such as transition to university or employment. Nostalgia was stated as a reason to visit the coast in Jarratt and Gammon (2016), much like for our own participants, which leads to the hypothesis that the coast facilitates coping with life transitions through the experience of nostalgia. Further research comparing the effect of the coast on different age groups is needed to test this hypothesis.

Just like with awe, the experience of nostalgia at the coast was sometimes expressed more ambivalently. More recent studies demonstrate that nostalgia is an ambivalent, but predominantly positive, emotion (Wildschut et al., 2006; Sedikides and Wildschut, 2016). Although most of our participants indicated feeling positive affect while recalling memories of the coast, other participants expressed a feeling of loss. This feeling of loss is what differentiates nostalgia from mere reminiscence, which is solely a positive form of recollection (Jarratt and Gammon, 2016).

The noted ambivalence in awe and nostalgia could potentially explain their positive influence on well-being. Indeed, studies have found that ambivalent emotions are more likely to support the fulfillment of psychological needs such as the need for autonomy and relatedness (Moss and Wilson, 2015) and to boost adaptive coping styles and resilience to stress (Braniecka et al., 2014). These findings are based on the coactivation model of Larsen et al. (2003) which stipulates that “taking the good with the bad” enables a person to confront adversity and to transform it into an advantage by engaging with the stressor and making meaning out of it. The bittersweet memory recounted by Anna could very well be an example of this very coping process. The present study therefore puts forward the hypothesis that the coast generates experiences of awe and nostalgia, which in turn enable coping with various life stressors, and ultimately benefit well-being.

Taking a look at the content of participants’ memories evoked by the coast is important to further understand this experience of nostalgia. Most of the memories were centered around a social context, which is consistent with studies portraying nostalgic narratives as often involving social interactions (Wildschut et al., 2006). Nostalgia has also been found to feature momentous events (Wildschut et al., 2006), such as the memory of swimming in the sea during a storm, described by Noa. Interestingly, other elements in nostalgic memories that are not prominently found in the literature were described in our interviews. These elements reflected for example different seasons at the coast (wintertime and summertime) and their associated natural phenomena, as well as simple daily routines at the beach. Therefore, beyond providing the opportunity to create important social memories, the coast can also elicit nostalgia through symbolic interactions with its natural elements.

#### Emotion Regulating Strategies

The fourth theme present in the interviews we conducted relates to emotion regulating strategies. Emotion regulation has been defined as “processes by which individuals influence which emotions they have, when they have them, and how they experience and express these emotions” (Gross, 1998, p. 275). Several emotion regulating strategies that are considered as adaptive have been described by our participants in relation to the coast. Although emotion regulation has been found to be positively associated with nature and to predict nature’s restorative outcomes (Johnsen, 2013), to the best of our knowledge, no studies have specifically evaluated emotion regulation in the context of coastal environments.

A first emotion regulating strategy refers to reflecting on life, problems, and emotions while being at the coast. Reflection has been suggested to contribute to active problem-solving and thereby lead to long-term positive outcomes (Arditte and Joormann, 2011). A previous study already found that exposure to nature increases the ability to reflect on a life problem (Mayer et al., 2009). A second strategy, or “family of strategies,” involves meaning-making processes such as acceptance and positive reappraisal, for which numerous psychological benefits have been found (Hayes et al., 1999; Gross and John, 2003). For example, reflection led some participants to be able to accept difficult situations or events, such as Arne accepting the possible “mistakes” he made, often in the context of his autism spectrum disorder (ASD). Other participants displayed positive reappraisal by putting things in perspective, namely to release the stress associated with things considered more trivial. These types of meaning-making processes could be partly due to the experience of awe and nostalgia triggered by the coast, as awe is associated with more focus on the “bigger picture” and nostalgia is linked with redemptive narratives (Wildschut et al., 2006; Piff et al., 2015). A third emotion regulating strategy refers to participants being able to let go or exteriorize emotions while being at the coast. Participants described being able to get a breath of fresh air, clear their mind, and let go of certain thoughts and emotions. This coincided with a deeper awareness of the present moment, and of feeling more connected to one’s surroundings. Other participants expressed using the coast for emotional exteriorization that ultimately enables them to calm down and have enough energy to move forward. This can be viewed as the opposite of suppression of emotional expression, which is considered as a risk factor for psychological distress (John and Gross, 2004).

If we take a closer look into these emotion regulating strategies, we notice that some of them resemble components of mindfulness. Mindfulness is considered as the nonjudgmental acceptance of thoughts, feelings, and sensations, while keeping a centered awareness of the present moment (Kabat-Zinn, 1990). This is in line with the strategies of acceptance and letting go described by the participants. We are therefore led to the hypothesis that the coast triggers emotional and cognitive processes that facilitate the practice of mindfulness, which could further enhance the benefits of well-being at the coast. Indeed, a study by Nisbet et al. (2019) demonstrates that practicing mindfulness while walking along a canal led to stronger connectedness to nature and reduced negative affect, compared to walking along the same path without mindfulness or walking indoors. Their exploratory analyses also revealed that mindfulness led to a higher experience of awe. Future research should therefore aim to replicate this effect for coastal environments.

The final subtheme identified within emotion regulating strategies involves seeking experiences of peak flow. Although emotion regulation and peak flow are independent constructs, it can be argued that they are positively interrelated (Tavares and Freire, 2016). Experiences of peak flow, typically arising during physical activity, are characterized by a feeling of intense satisfaction, that occurs when a person considers their level of skill as sufficient to meet the perceived challenges of that activity (Pomfret and Bramwell, 2016). An example of such an experience was described by Noa; these experiences have been previously reported to take place at the coast (Bell et al., 2015). In addition to triggering peak flow, the coast also appears to facilitate positive stimulation without directly overwhelming our participants with high sensitivity. A study by Caddick et al. (2015) displays similar findings as it explored the effect of surfing on the well-being of combat veterans experiencing posttraumatic stress disorder (PTSD). Their participants expressed that the sensory experience of surfing at the coast enables them to escape from their usual ruminative thinking that typically overwhelms them. It would be beneficial to conduct further research into the benefits of peak flow experiences and positive stimulation at the coast for individuals with mental health disorders such as ASD or PTSD.

#### Coast as a Safe Haven

The fifth and final theme that emerged from the interviews was the coast as a safe haven. The theme essentially demonstrates that for all participants, the coast represents a safe place where they can feel emotional and vulnerable and have respite from the stressors and pressures of daily life. Participants even described a certain ability to disconnect from reality, by becoming lost in their thoughts. This strongly reflects the concept of “being away” within the attention restoration theory, defined by psychological distance from one’s usual thoughts and concerns that require directed attention (Kaplan and Kaplan, 1989). There therefore seems to be a shift in the content and the type of processing of thoughts, with the coast enabling more “reverie,” or in other words more unconscious musings over one’s life for example (Jarratt and Gammon, 2016).

In relation to this, Noa described a sense of timelessness at the coast, which has been previously linked to the experience of nostalgia, due to the contrast between the permanence of the coast and the fast-paced modern world (Jarratt and Gammon, 2016; Jarratt and Sharpley, 2017). The study of Rudd et al. (2012) demonstrates that the emotion of awe is also found to be associated with an impression of timelessness, as awe increased perceived time availability, which subsequently led to greater life satisfaction. The coast therefore perhaps expands the perception of time through the experience of awe and nostalgia, thereby potentially counteracting “time famine.” Time famine refers to the feeling of having too much to do and not enough time to do it (Perlow, 1999). Investigating this mechanism would be beneficial considering that time famine has been shown to have negative health effects, and is possibly associated with burnout (Lehto, 1998; see also Bell et al., 2017).

An additional aspect of the coast as a safe haven relates to the sense of freedom that some participants associate with the coast. Indeed, the COVID-19 pandemic appeared to strengthen this sense of freedom by providing an accessible open-space with a diverse landscape. This is consistent with findings of a positive relationship between access to the coast and well-being during the first-wave lockdown in Belgium (Severin et al., 2021).

To understand why the coast represents a safe haven for our participants, we can link this to the representation of the coast as “home.” This representation was also demonstrated by Jellard and Bell (2021), who stated that the coast could be considered as a “home away from home” (Gardner, 2011), enabling a feeling of ease, stability, and connectivity with others who share that same space. Moreover, our participants’ representation of the coast strongly reflects the notions of place attachment (i.e., the emotional bond to a place) and place identity (i.e., the cognitive identification to a place; Jorgensen and Stedman, 2001). Place attachment has been linked to numerous psychological benefits, as well as the emotion of nostalgia (Scannell and Gifford, 2017). Additionally, Korpela (1989) argues that place identity is partly formed by processes of emotion- and self-regulation. For example, the ability to reflect in one’s favorite place can help maintain one’s sense of coherence and self-esteem, which, in turn, can enable the place to have restorative qualities (Korpela and Hartig, 1996). Further research should aim to assess to what extent attachment or familiarity with the coast generates this representation of safe haven and/or whether there are other mechanisms at play.

One example of such a mechanism could be the opportunity to be alone. Most urban cities in Belgium do not have natural spaces that offer the same level of openness such as the coast. In exception to during summertime, the vastness of the coast therefore allows one to be alone, or to the very least, feel alone. Being alone at the coast was appreciated by most of the participants and appeared to facilitate emotional restoration and emotion regulating strategies. The limited presence of other people most likely enables participants to focus their attention to their natural surroundings and/or to themselves.

### Limitations, Future Directions, and Implications

The present study was conducted with the use of the well-validated approach of interpretative phenomenological analysis, that enabled us to explore the lived emotional experience generated at the coast for coastal residents. There are nonetheless certain limitations present in the study. The first limitation arises from implementing the interviews *via* videoconferencing and not physically face to face. This sometimes led to technical hiccups, which possibly formed miscommunications. Additionally, videoconferencing created a different interview atmosphere, in which, on the one hand, some participants potentially had more difficulty opening up and building a relationship of trust with the interviewer. On the other hand, for other participants, this may have facilitated vulnerable exposure due to the psychological distance created by an online interview (Roesler, 2017).

Furthermore, in exception to Noa, our sample consisted of participants that were students or were permanently employed, indicating a minimum amount of socioeconomic stability. Although our participants expressed a number of stressors in their lives, it would be interesting to analyze how groups that undergo a higher degree of stress, such as patients in a rehabilitation center, or individuals with a lower socioeconomic status, experience their emotions at the coast. Although the association between coastal proximity and mental health is shown to be stronger for those with low household income (Garrett et al., 2019), the mediating role of emotional factors for this group remains unknown. Nonetheless, two of our participants are highly sensitive, with one of them having autism spectrum disorder. Recruiting participants with these characteristics was not intended but their experiences helped bring more insight to our analysis, in terms of the function of the coast in emotionally coping with such characteristics.

An important element that should also be taken into account is the contextual influence of the COVID-19 pandemic. During the time period of the interviews, Belgium was experiencing an intense second-wave of COVID-19 infections, along with a number of governmental measures such as closing of nonessential stores and restaurants, a curfew, and restricted social contacts. This second-wave seemed to particularly affect young adults as a series of health surveys demonstrated a rise in anxiety, depression, and dissatisfaction with life for those aged between 18 and 24 years old, compared to the first-wave of COVID-19 infections (Berete et al., 2020). The interpretation of our findings is therefore dependent on this context. For example, several participants expressed the negative impact of the pandemic on their daily routines, vacation jobs, and social interactions. In parallel, participants reported frequently visiting the coast during the lockdown. Considering the overall impact of the pandemic on mental health, it is possible that the prominence of engaging in emotion regulation at the coast was enhanced during this particular time period. Additionally, the pandemic enabled more opportunities to be alone, which perhaps reinforced the notion of the coast as a safe haven.

Finally, our findings cannot be generalized to the general population due to the qualitative focus on the subjective experience of our sample of young coastal residents. Nonetheless, our findings represent various hypotheses that should be tested with quantitative methods, such as the elicitation of awe and nostalgia and the use of adaptive emotion regulating strategies at the coast. The interactions between these different emotional processes should also be further investigated.

In addition to these future directions, several theoretical and practical implications can be taken out from our findings. On a theoretical level, we can hypothesize that, due to their beneficial nature, the stated emotions and related emotion regulating strategies elicited by the coast may play a positive role on the well-being of our participants. This could thus indicate an indirect relationship between the coast and well-being. Moreover, our findings demonstrate that coastal residents can experience similar emotional processes as coastal visitors/tourists, such as the emotions of awe and nostalgia. However, the feeling of safety at the coast is perhaps unique to coastal residents, due to their familiarity with the coast. Our study therefore enriches the literature with a specific look into emotional mechanisms in the relationship between the coast and well-being for coastal residents.

On a practical level, we suggest that we aim to preserve the factors that facilitate the beneficial emotional experiences highlighted in our study. For example, we should promote physical activity at the coast, such as watersports, that enable experiences of peak flow or emotional restoration. We should also promote social interactions but only to a reasonable extent. Based on our interviews, experiencing nostalgia was partly dependent on important social memories created at the coast; however, the opportunity to be alone was also associated with various adaptive emotion regulating strategies. Additionally, mass tourism during the summer has a negative impact on the coast’s restorative qualities. Efforts should therefore be made to limit overcrowding, such as optimizing spatial distribution (Basterretxea-Iribar et al., 2019) or instating reservation systems to access the beach. The emotion of awe also depends on natural characteristics such as openness and aesthetic beauty; these elements should therefore be safeguarded, such as by reducing littering at the coast.

Finally, investigating the role of emotional processes triggered by the coast on well-being can bring insight into possible solutions in the prevention of psychological distress and support of mental health. Developing the use of mindfulness at the coast with the elicitation of awe could for example potentially benefit coping with burnout or mood disorders or even other conditions undermining people’s well-being. Furthermore, the findings represented in our study could lead to important opportunities for contexts other than mental health. For example, the emotional and restorative experience of the coast could be recalled and utilized to promote training of emotional intelligence. In short, emotional intelligence skills can be divided into four abilities, namely the ability to perceive emotions, to use emotions to facilitate thought, to understand emotions, and to manage emotions (Mayer et al., 2004). Developing these skills has been found to positively affect academic performance, social interactions, and organizational behavior (Mayer et al., 2004). Young adults could particularly benefit from these skills, considering that they experience important transitions within these educational, social, and work contexts (Arnett et al., 2014).

## Conclusion

The present study demonstrates that young coastal residents can experience complex emotional processes at the coast, namely the ambivalent emotions of awe and nostalgia, as well as emotional restoration. These emotions appear to be facilitated by the coast’s multisensory and symbolic qualities. Furthermore, the coast is featured as a safe place that allows participants to engage in adaptive emotion regulating strategies and to distance themselves from daily life stressors. These findings indicate a potential benefit of these emotional processes on the well-being of our participants. We therefore argue that the emotional mechanisms highlighted in our study should be considered as possible contributors to the coast’s therapeutic potential, alongside the putative physical, social, and cognitive mechanisms.

## Data Availability Statement

The raw data supporting the conclusions of this article will be made available by the authors, without undue reservation.

## Ethics Statement

The studies involving human participants were reviewed and approved by the Ethical Committee of the Faculty of Psychology and Educational Sciences of Ghent University. The patients/participants provided their written informed consent to participate in this study.

## Author Contributions

MS contributed to the conceptualization, methodology, data analysis, and writing of the manuscript. LL and EN wrote their master thesis based on the present study and took part in the conceptualization, methodology, data collection, and data analysis. FR, GE, and AB were involved in conceptualization, supervision, and review and editing of the manuscript. AB also supervised the data analysis. All authors contributed to the article and approved the submitted version.

## Conflict of Interest

The authors declare that the research was conducted in the absence of any commercial or financial relationships that could be construed as a potential conflict of interest.

## Publisher’s Note

All claims expressed in this article are solely those of the authors and do not necessarily represent those of their affiliated organizations, or those of the publisher, the editors and the reviewers. Any product that may be evaluated in this article, or claim that may be made by its manufacturer, is not guaranteed or endorsed by the publisher.

## Footnotes

^1^ https://osf.io/tm7pj

^2^Each participant’s name has been replaced by a pseudonym to protect confidentiality.
